# Potential role of creatine as an anticonvulsant agent: evidence from preclinical studies

**DOI:** 10.3389/fnins.2023.1201971

**Published:** 2023-06-29

**Authors:** Eman A. Alraddadi, Abdulrahman M. Khojah, Faisal F. Alamri, Husun K. Kecheck, Wid F. Altaf, Yousef Khouqeer

**Affiliations:** ^1^Department of Basic Sciences, College of Science and Health Professions, King Saud bin Abdulaziz University for Health Sciences, Jeddah, Saudi Arabia; ^2^King Abdullah International Medical Research Center, Jeddah, Saudi Arabia; ^3^College of Medicine, King Saud bin Abdulaziz University for Health Sciences, Jeddah, Saudi Arabia

**Keywords:** creatine, epilepsy, seizure, antioxidant, mitochondrial dysfunction, anticonvulsant

## Abstract

Epilepsy is one of the most common neurological disorders affecting people of all ages representing a significant social and public health burden. Current therapeutic options for epilepsy are not effective in a significant proportion of patients suggesting a need for identifying novel targets for the development of more effective therapeutics. There is growing evidence from animal and human studies suggesting a role of impaired brain energy metabolism and mitochondrial dysfunction in the development of epilepsy. Candidate compounds with the potential to target brain energetics have promising future in the management of epilepsy and other related neurological disorders. Creatine is a naturally occurring organic compound that serves as an energy buffer and energy shuttle in tissues, such as brain and skeletal muscle, that exhibit dynamic energy requirements. In this review, applications of creatine supplements in neurological conditions in which mitochondrial dysfunction is a central component in its pathology will be discussed. Currently, limited evidence mainly from preclinical animal studies suggest anticonvulsant properties of creatine; however, the exact mechanism remain to be elucidated. Future work should involve larger clinical trials of creatine used as an add-on therapy, followed by large clinical trials of creatine as monotherapy.

## 1. Introduction

Epilepsy is one of the most prevalent chronic neurological disorders affecting more than 70 million people worldwide (Singh and Sander, 2020). Additionally, a greater number of people experience seizure at least once in their lifetime, resulting in significant social and public health burden (Hauser and Beghi, 2008). The annual cost for epilepsy in the United States alone is estimated to be approximately 12.5 billion dollars (Begley et al., 2000). It affects people of all ages; however, it is especially common in older people (over 75 years) and children under the age of 2 (Thijs et al., 2019).

Epilepsy is a disorder of the brain generally characterized by a lasting predisposition to generate spontaneous epileptic seizures (Thijs et al., 2019). A seizure is a brief, sudden, and excessive discharge of electrical activity of cerebral neurons leading to variety of events, including loss of consciousness, abnormal movements, atypical or odd behavior, and distorted perceptions that are of brief duration but may recur if untreated (Falco-Walter, 2020). This condition can be manifested either due to genetic predisposition or acquired from acute brain insult such as traumatic brain injury (TBI), stroke, and central nervous system (CNS) infections, however, in some cases the cause is unknown (Balestrini et al., 2021). The term epilepsy encompasses a huge variety of syndromes with no single etiology, and has numerous neurobiological, psychological, cognitive, and social consequences affecting the patients quality of life (Fisher et al., 2005).

The main goal in the management of epilepsy is to terminate seizure as early as possible with minimal side effects and to achieve seizure remission, which will likely reduce morbidity and decrease the risk of premature mortality (Devinsky et al., 2016). Anti-epileptic drugs (AEDs) are the main form of symptomatic treatment for patients with epilepsy (Sills and Rogawski, 2020). Pharmacological targets of the current available AEDs can be categorized into three main groups: (1) modulation of voltage-gated ion channels, including sodium, potassium, and calcium channels; (2) enhancement of γ-aminobutyric acid (GABA)-mediated inhibition by targeting GABA receptors, transporters, or enzymes involved in its metabolism; (3) inhibition of synaptic excitation mediated by glutamate receptors (Sills and Rogawski, 2020). Despite the availability of over 25 medications for epilepsy, in which most were approved over the last 30 years, the current drugs are effective in only about 65%–70% of individuals and as many as 30% of patients are considered to have refractory epilepsy even with the use of a combination of drugs (Kwan et al., 2011). One possible explanation of the high percentage of refractory epilepsy is that most of the AEDs available today were discovered by screening or by serendipity and their mechanisms of actions were only identified after their discovery or clinical approval (Loscher et al., 2020). On the other hand, only few AEDs discoveries were the result of rational, target-based strategies and were biased toward agents that modulate only a single pathology in epilepsy, that is, imbalance of GABAergic inhibition and glutamatergic excitation (Loscher et al., 2020). However, the view that epilepsy is solely due to an imbalance between excitatory and inhibitory transmission in the brain ignores the complexity of the alterations within these neurotransmitters systems and the pathological changes that occurs in the brains of affected individuals (Lukawski and Czuczwar, 2021). This highlights the importance of identifying novel targets for the development of more effective therapeutics.

### 1.1. Involvement of mitochondrial dysfunction in epilepsy

There is growing evidence pointing to a strong involvement of mitochondrial dysfunction and bioenergetic impairment in the development of seizure and epilepsy. First evidence arises from the knowledge that around 25% of patients with inherited mitochondrial respiratory chain disorders often develop epilepsy (Rahman, 2018; Chan et al., 2019). On the other hand, one-third of individuals with refractory epilepsy were found to have biochemical evidence of mitochondrial dysfunction (Parikh et al., 2008). In addition, neurological conditions known to trigger epilepsy, such as TBI and stroke, have mitochondrial dysfunction as a central component in their pathology (Fordington and Manford, 2020; Galovic et al., 2021).

**Figure 1:**
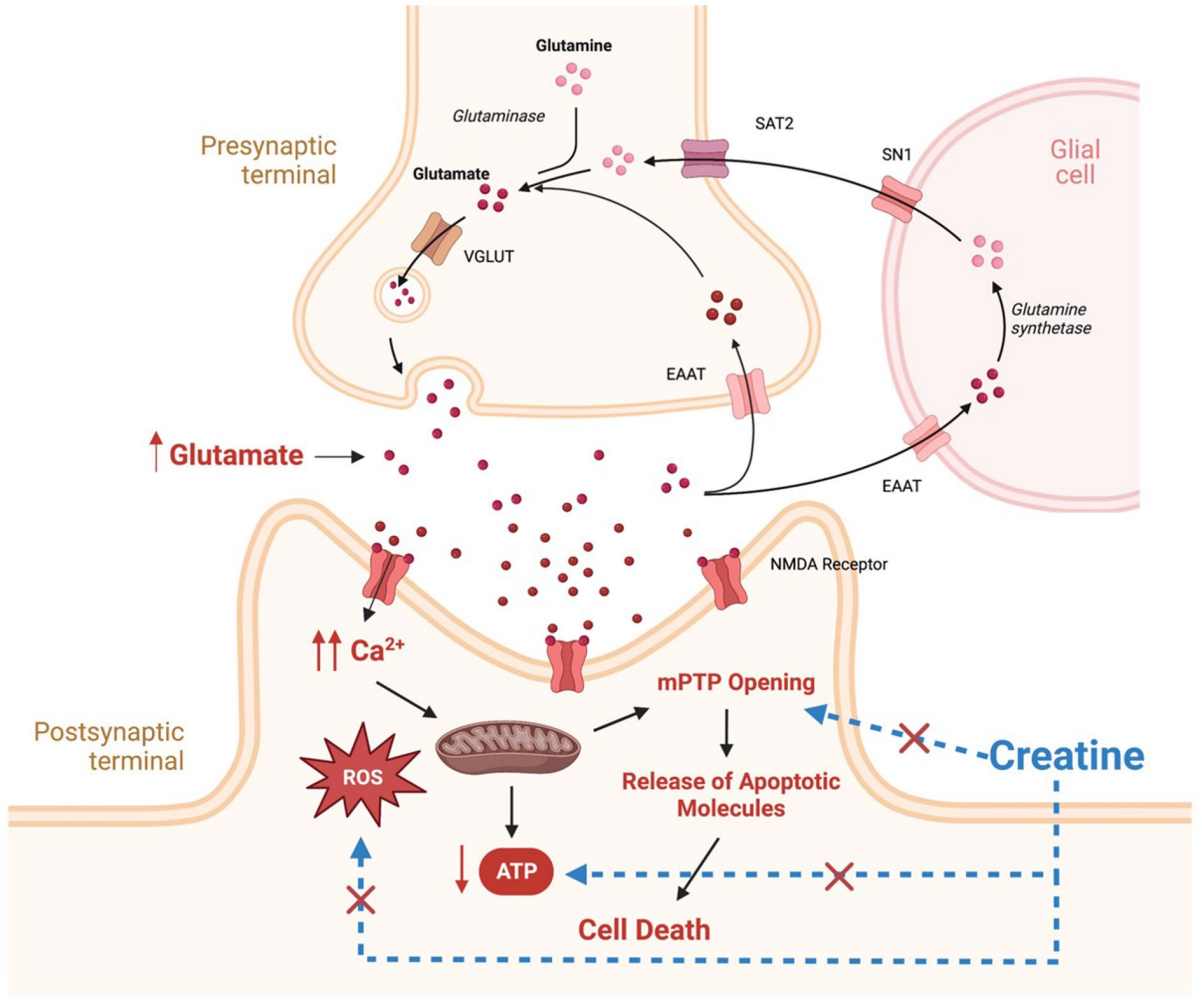
Potential mechanisms involved in the pathogenesis of epilepsy and potential targets for creatine neuroprotective effects. Therapeutic targets for creatine supplementation are denoted by blue dashed lines. SAT2, system A transporter 2; SN1, system N transporter 1; EAAT, excitatory amino acid transporter; VGLUT, vesicular glutamate transporter; NMDA, N-methyl-D-aspartate; ROS, reactive oxygen species; MPTP, mitochondrial permeability transition pore. This figure is created with BioRender.com.

Early pioneering experimental studies demonstrated that energetic substrates such as glucose and adenosine triphosphate (ATP) are reduced during seizure activity induced by chemical agents in rodents as experimental model of acute seizure (Sacktor et al., 1966; King et al., 1967; Sanders et al., 1970). In fact, the concentration of ATP in the brain is uniformly and significantly decreased even prior to the onset of seizure (Sanders et al., 1970). In addition, significant depletion of cerebral ATP is observed after initiation of seizure. The apparent depletion of cerebral ATP has been attributed to increased ATP utilization secondary to seizure, which presumably exceeds the rate of ATP synthesis (Klein and Olsen, 1947). Later, these findings were confirmed in recent studies on human brain specimens from patients with temporal lope epilepsy. In these studies, impairment in mitochondrial function such as reductions in the activity of electron transport chain (ETC) enzymes, specifically complex I activity, and reduction of N-acetyl aspartate levels were found in the hippocampal region of the brain (Kunz et al., 2000; Vielhaber et al., 2008). In addition, it was demonstrated that epileptogenic injury impairs hippocampal bioenergetics, as assessed by a decrease in the ATP/adenosine diphosphate (ADP) ratio, which could reflect an increase in the utilization of ATP during seizure activity (Wasterlain et al., 2010; Kovac et al., 2012). Moreover, a decrease in maximal respiratory and reserve capacities was also found in studies of animal models of epilepsy, reflecting a decline in the ability to produce ATP on demand (Rowley et al., 2015). This suggests that bioenergetic impairment plays a significant role in seizure formation and contributes to altered neuronal excitability (Figure 1).

In addition to energy failure, abnormally elevated levels of glutamate and increased activation of its receptors, result in intracellular calcium accumulation and increased production of reactive oxygen species (ROS) (Figure 1; Kovac et al., 2017). Elevated calcium and ROS levels are known to be potent triggers for mitochondrial permeability transition pore (mPTP) opening, leading to mitochondrial swelling and cytochrome C release, subsequently, activating cell apoptosis cascade (Figure 1; Kovac et al., 2017). In addition, Excess ROS production has deleterious effects on mitochondrial components such as mitochondrial DNA, mitochondrial proteins, lipids, and respiratory chain functions, leading to insufficient energy supply for cells, triggering more ROS generation and disturbances in calcium homeostasis, thus creating a mitochondrial free radical vicious cycle of injury (Yang et al., 2020). The net result is increased neuronal excitability, seizure susceptibility, and vulnerability to additional stress and neuronal loss (Yuen et al., 2018).

As a result, several research studies have begun to focus on examining anticonvulsant potential of compounds that could improve overall brain bioenergetics and mitochondrial function (Reid et al., 2014; Alqahtani et al., 2020). In particular, there has been increasing interest in the potential anticonvulsant properties of creatine, a key player in cellular energy metabolism. Hence, in this review, we will shed the light on the beneficial cellular effects of creatine, rational and goals of exogenous creatine supplementations, and we will discuss currently available preclinical evidence pertaining to its anticonvulsant effects.

## 2. Creatine

Creatine, is a nitrogenous guanidine compound that is both synthesized endogenously from amino acids and found exogenously in various protein based food sources such as meat, fish, and nuts (Maughan, 2018). It was first isolated from animal skeletal muscle in 1832, and since then, creatine has been one of the most extensively studied dietary supplements (Maughan, 2018). In the early 1990s, studies started to document beneficial ergogenic effects of creatine on exercise performance and muscle mass, particularly in short, high-intensity exercises (Balsom et al., 1993; Williams and Branch, 1998; Kreider, 2003; Hall and Trojian, 2013). Creatine is now widely used as an ergogenic agent among recreational and professional athletes with a global market of more than 400 million United States dollars annually (Butts et al., 2018).

### 2.1. Importance of creatine to the brain

**Figure 2:**
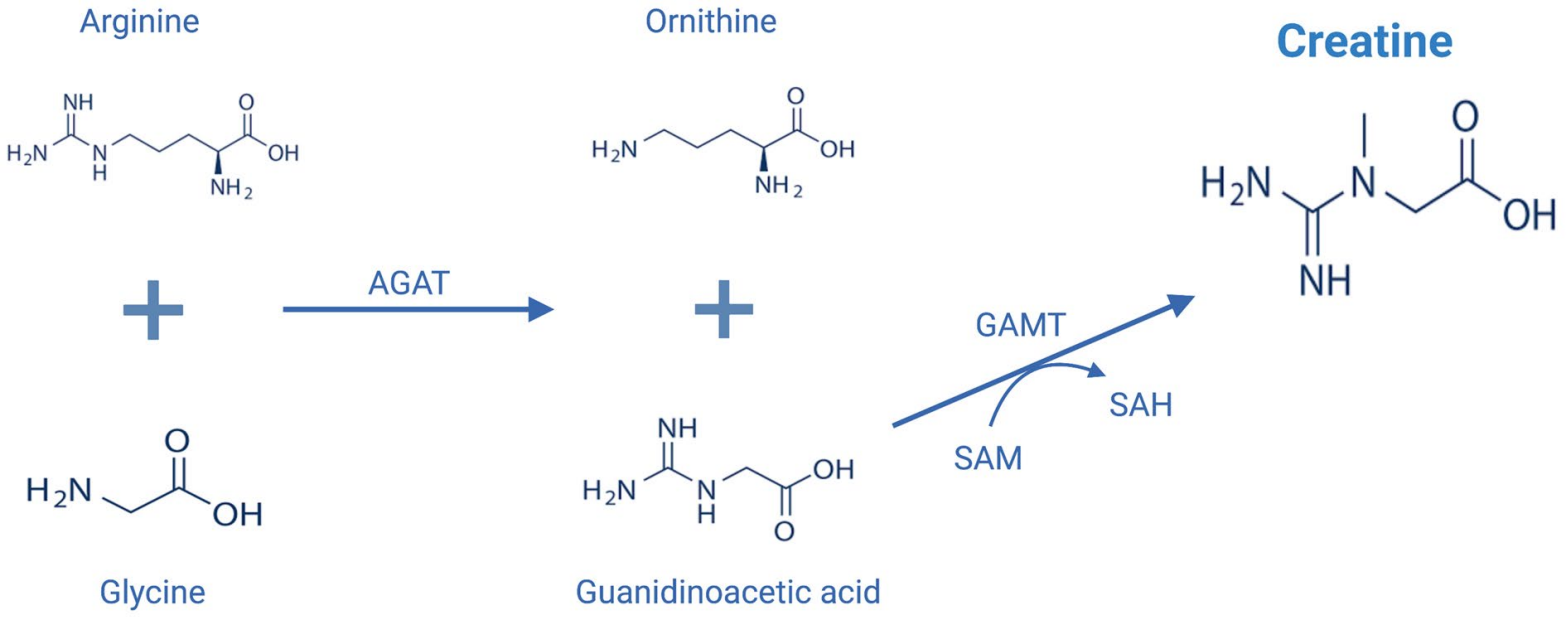
Endogenous creatine biosynthesis pathway. AGAT, L-arginine:glycine amidinotransferase; GAMT, S-adenosyl-L-methionine:N-guanidinoacetate methyltransferase; SAM, S-adenosylmethionine, SAH: S-adenosylhomocysteine. This figure is created with BioRender.com.

Endogenous synthesis of creatine mainly takes place in the kidney and liver by the action of two key enzymes, L-arginine:glycine amidinotransferase (AGAT) and S-adenosyl-L-methionine:N-guanidinoacetate methyltransferase (GAMT) (Figure 2; Wyss and Kaddurah-Daouk, 2000). In comparison to skeletal muscles, only a small amount of total creatine is found in the brain, however, the brain is an energetically demanding organ and accounts for approximately 20% of body energy consumption despite accounting for only 2% of total body mass (Forbes et al., 2022). The mammalian brain was found to express both AGAT and GAMT enzymes and can synthesize creatine to a limited degree, but brain creatine levels are primarily maintained by active transport via a sodium- and chloride-dependent creatine transporter (CRT1) (Snow and Murphy, 2001; Ohtsuki et al., 2002). Evidence supporting the importance of blood brain barrier (BBB) creatine transport comes from studies of patients with genetic mutations within the CRT1 transporter. Individuals with CRT1 deficiency, caused by mutations in the SLC6A8 gene, have reduced levels of creatine in the brain despite normal functioning of AGAT and GAMT (Fons and Campistol, 2016). This reduction results in various neurological symptoms, including epilepsy, severe intellectual disability, autism, and motor dysfunction (Fons and Campistol, 2016). Those patients seem to be unresponsive to exogenously administered creatine even when high doses are administered (Fons and Campistol, 2016). On the other hand, AGAT and GAMT deficient patients who have reduced ability for *de novo* synthesis of creatine, can be successfully treated with oral creatine supplementation, although very high doses of creatine are used, and the replenishment of cerebral creatine takes months (Ganesan et al., 1997). This emphasizes the importance of exogenously administered creatine as determinants of brain levels of creatine and has increased interest in potential benefits of creatine supplementation in the brain.

### 2.2. Cellular effects of creatine

Within the cell, creatine plays a key role in cellular energy metabolism especially in tissue with high and fluctuating energy demands (e.g., muscle, heart, and brain) (Wallimann et al., 1998). It is reversibly phosphorylated to phosphocreatine (PCr) by creatine kinase (CK), and can thus either be utilized as PCr to generate energy by donating its phosphoric group to ADP to convert it to ATP or alternatively stores energy in a more stable form and up to 10 times the amounts available in the ATP pool that may be used when needed in a pulsed or fluctuating manner (Wallimann et al., 1998). This system is involved in energy metabolism in three ways that have been extensively reviewed elsewhere (Wallimann et al., 2011): (1) It functions as a “temporal energy buffer,” maintaining an adequate ATP/ADP ratio during increased energy demands. (2) It also functions as an “ATP shuttle,” transferring ATP from its production site (i.e., mitochondria) to areas of utilization (e.g., cytoplasm, membrane ATPase). In this manner, creatine receives the phosphate group from ATP in the mitochondria and is converted to PCr. It then diffuses to the periphery of the cell providing ATP where it is needed. (3) Lastly, it plays an important compensatory role under pathological conditions of increased energy demands or reduced energy production (Wallimann et al., 2011).

In addition to helping maintain cellular energetics, several additional beneficial effects of creatine have been suggested and supported by evidence from recent studies. Creatine supplementation was demonstrated to have antioxidant properties via a mechanism involving direct scavenging of ROS, or alternatively, reducing the production of mitochondrial ROS indirectly by improving mitochondrial respiration and cellular energy capacity (Lawler et al., 2002; Guidi et al., 2008; Sestili et al., 2011). Moreover, A direct anti-apoptotic effect of creatine has been reported by studies on which creatine supplementation prevented mPTP opening, which is an early event in apoptosis (O’Gorman et al., 1997; Dolder et al., 2003). Together, the documented beneficial effects of creatine on cellular respiration and mitochondrial function have increased interest in the use of creatine supplementation in neurological conditions in which mitochondrial dysfunction is a central component in its pathology.

### 2.3. Applications of creatine supplements

**Figure 3:**
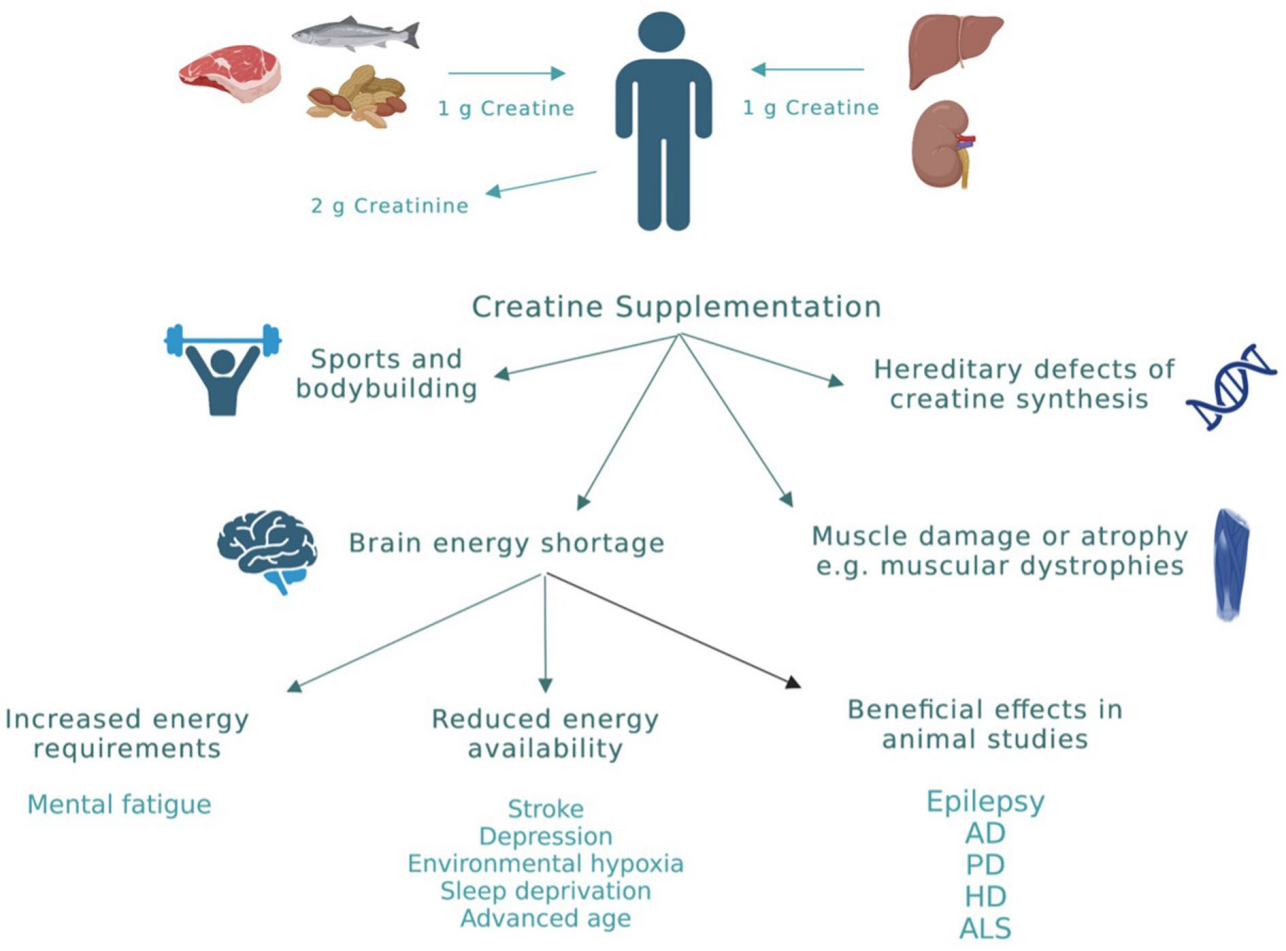
Creatine sources and applications. Physiologically, the daily loss of creatine due to its break down to creatinine is around 2 g. This amount is replenished by endogenous synthesis (≈ 1 g/daily) and through dietary creatine (≈ 1 g/daily). However, in cases of high-energy demands or reduced energy production, exogenous creatine supplementation is required to produce beneficial effects. Clinical conditions in which there is evidence for beneficial effects of creatine from human studies are highlighted in this figure. AD, Alzheimer’s disease; PD, Parkinson’s disease; HD, huntington’s disease; ALS, amyotrophic lateral sclerosis. This figure is created with BioRender.com.

Among all biochemical reactions that the cell uses to generate ATP, the creatine system is the quickest and most efficient in buffering the levels of ATP especially at times of increased energy demands (Sahlin and Harris, 2011). This explains the increased interest over the past decade in creatine supplementation in various physiological and pathological conditions. Physiologically, the daily loss of creatine due to its break down to creatinine is around 2 g. This amount is replenished by endogenous synthesis and through dietary creatine (Figure 3; Kreider et al., 2017). However, in cases of high-energy demands or reduced energy production, large amounts of creatine are required to produce beneficial effects that can only be obtained through exogenous supplementation (Kreider et al., 2017). To date, creatine supplementation, mainly in the form of creatine monohydrate (CM), has been exploited mainly by athletes and bodybuilders as a nutritional supplement to improve performance. However, there has been an emergence of research investigating the impact of creatine supplementation in a variety of disorders where cellular energy production is compromised. The advantage of increasing tissue creatine and PCr to supraphysiological levels is to augment the metabolic capacity of the tissue. The net result is improving cellular energy state (Greenhaff et al., 1993), facilitating intracellular energy transport (Saks et al., 1978), improving the efficiency of cellular energy utilization (van Leemputte et al., 1999), and stimulating mitochondrial respiration (Kay et al., 2000). Clinical conditions in which there is strong rational and evidence from human studies supporting the effectiveness of creatine supplementation are shown in Figure 3 and include: (1) Conditions where endogenous creatine is decreased due to inborn errors in genes responsible for creatine synthesis. (2) Muscle-related disorders in which there is muscle damage or atrophy, such as muscular dystrophies. (3) Conditions of brain energy shortage, either due to increased energy requirements or reduced energy availability (Balestrino and Adriano, 2019).

In addition to the above-mentioned clinical conditions, there is increased interest in the use of creatine in neurological conditions in which energy depletion, oxidative stress, and mitochondrial dysfunction are central components in their pathology (Figure 3). To date, creatine supplementation has been shown to be neuroprotective in many cellular and animal models of neurodegenerative diseases including Huntington’s disease (HD), Alzheimer’s disease (AD), Parkinson’s disease (PD), and amyotrophic lateral sclerosis (ALS) (Figure 3; Klein and Ferrante, 2007; Adhihetty and Beal, 2008; Gualano et al., 2010). The exact molecular mechanism by which creatine exert its neuroprotective effect is not clearly understood. However, several potential mechanisms of neuroprotection of creatine have been suggested and a combination of more than one mechanism is more likely to cause the significant neuroprotection seen with creatine administration in rodents studies. Some of the reported mechanisms of neuroprotection of creatine include buffering intracellular energy reserves and improving mitochondrial function, stabilizing mPTP, and antioxidant activity (Beal, 2011). Although there is increasing preclinical evidence supporting the role of creatine in the prevention and/or treatment of many neurological disorders, the potential benefit of creatine in convulsive disorders has not been clearly defined. Given the beneficial effects of creatine on mitochondrial function, oxidative stress, and energy production, and the fact that these mechanisms are known to be contributing factors in the development of epilepsy, research is now focusing on the possible neuroprotective and anticonvulsant properties of creatine in convulsive disorders. Figure 1 shows the pathological mechanisms involved in the development of epilepsy and the potential targets of creatine treatment. Previously, it was shown that exogenous creatine supplementation protects against seizure induced by methylmalonic and glutaric acid, reduces lactate production and oxidative damage, suggesting an anticonvulsant and antioxidant role of this compound in experimental models of inborn errors of metabolism that present as epileptic phenotypes (Royes et al., 2003, 2006; Magni et al., 2007).

### 2.4. Anticonvulsant effects of creatine supplementation

The importance of creatine for the epileptic brain is clearly demonstrated in previous studies exploring the metabolic changes of creatine and PCr during seizure (Petroff et al., 1984; DeFrance and McCandless, 1991; Lee et al., 2019). Previously, it was shown that during seizure activity in rodents, ATP and PCr levels are significantly reduced indicating an increase in energy expenditure with resultant lowering of key energy metabolites in the hippocampus (Petroff et al., 1984; DeFrance and McCandless, 1991; Lee et al., 2019). On the other hands, rodents and human studies revealed increases in the concentrations of creatine in the hippocampus during seizure activity (Connelly et al., 1994; Gadian et al., 1994; Lee et al., 2019). These phenomena could be explained by the complementary relationship between creatine and PCr, in which levels of creatine are increased to compensate for the reductions in PCr. In this section, evidence of anticonvulsant effects of creatine supplementation from previously published studies in preclinical animal models will be discussed.

The effects of creatine supplementation in modulating seizure propensity were examined in different experimental animal models of seizure. A summary of the main findings from these animal studies is provided in Table 1. In a study by Shafaroodi et al. (2016), three mouse models were used to explore the role of creatine on seizures, intravenous pentylenetetrazol (PTZ), intraperitoneal PTZ, and electroshock models. PTZ injected rodents are widely used as a chemically induced acute seizure model. PTZ is a GABA-A receptor antagonist, it suppresses the function of inhibitory synapses leading to increased neuronal activity (Shimada and Yamagata, 2018). PTZ administration induces generalized tonic–clonic seizure and is useful for rapid screening of AEDs (Kandratavicius et al., 2014). PTZ-induced seizures lead to an increase in local cerebral glucose, a higher CK rate constant, increased ATP utilization, and higher ATP turnover (Bonan et al., 2000). Apart from PTZ seizure models, electroshock-induced seizure is another model for generalized tonic–clonic (grand mal) seizure and is universally employed today in anticonvulsant screening (Shafaroodi et al., 2016). In this study, acute (10 and 20 mg/kg, 60 min before PTZ) and sub-chronic (10 and 20 mg/kg, 5 days before PTZ) intraperitoneal creatine administration, revealed significant anticonvulsant effects and increased seizure threshold. In addition, acute creatine treatment, significantly reduced the frequency of seizure development following intraperitoneal PTZ administration and electroshock in mice (Table 1; Shafaroodi et al., 2016). Another study by Rambo et al., evaluated the effects of acute creatine administration (300 mg/kg orally) on intraperitoneal PTZ-induced electrographic, oxidative, and neurochemical alterations, and mitochondrial membrane potential in rat cerebral cortex (Rambo et al., 2013). They found that creatine treatment, 45 min prior to PTZ administration, increased the latency period for both the first myoclonic jerk and the first generalized tonic–clonic seizure and reduced time spent in generalized tonic–clonic seizure. In addition, creatine prevented the increase in electroencephalographic wave amplitude typically elicited by PTZ (Rambo et al., 2013). Moreover, creatine administration prevented PTZ-induced decreases in Na^+^/K^+^/ATPase activity as well as ATP and ADP levels in the cerebral cortex. The same dose of creatine also prevented the increase in xanthine oxidase activity and uric acid levels induced by PTZ. Lastly, creatine was shown to prevent PTZ-induced mitochondrial dysfunction resulting in increased mitochondrial membrane potential in the cerebral cortex (Table 1; Rambo et al., 2013). In this sense, it appears that creatine can maintain and improve both mitochondrial function and energy generation while reducing oxidative stress during excitotoxic processes such as seizures. Using the same rat seizure model, Rambo et al., examined whether physical exercise and creatine supplementation has additive anticonvulsant effects against PTZ-induced seizure (Rambo et al., 2009). In this study, 6 weeks of physical training or creatine supplementation (300 mg/kg, orally) decreased the duration of PTZ-induced seizures in rats, as measured by cortical and hippocampal electroencephalography and behavioral analysis. Importantly, the combination of physical training and creatine supplementation had additive anticonvulsant effects, since it increased the onset latency for PTZ-induced seizures and was more effective in decreasing seizure duration than physical training or creatine supplementation individually (Table 1; Rambo et al., 2009).

**Table 1:** A summary of the main findings of the effects of creatine supplementation in various animal models of epilepsy.

Study	Epilepsy model	Dosage and duration	Main findings
Shafaroodi et al. (2016)	Male albino mice: IV PTZ. IP PTZ. Electroshock.	IV PTZ experiment: Creatine (5, 10, 20, 40, and 80 mg/Kg) IP 60 min before IV PTZ (acute effect). Creatine (10 and 20 mg /Kg) IP for 5 days followed by IV PTZ on the 6^th^ day (sub-chronic effect). IP PTZ experiment: Creatine (10, 20 and 40 mg/Kg) IP 60 min before IP PTZ (acute effect). Electroshock experiment: Creatine (20, 40 and 80 mg/Kg) IP 60 min before electroshock (acute effect).	Increased the threshold of clonic seizures in IV PTZ model, significant anticonvulsant effect. Decreased the frequency of clonic seizure in IP PTZ model. Decreased the incidence of tonic seizures after electroshock.
Rambo et al. (2013)	Adult male Wistar rats: IP PTZ.	Creatine (300 mg/kg) orally 45 min prior to treatment with PTZ.	Prevented the increases in electroencephalographic wave amplitude. Increased myoclonic jerks and generalized tonic–clonic seizure latency period. Reduced time spent in generalized tonic–clonic seizure. Reduced oxidative stress markers.
Rambo et al. (2009)	Adult male Wistar rats: IP PTZ.	Creatine (300 mg/kg) orally with or without. physical exercise for 6 weeks.	Creatine decreased duration of PTZ-induced seizure. Combination of creatine and exercise had additive anticonvulsant effects.
Gerbatin et al. (2019)	Adult male Wistar rats: IP PTZ following TBI induction.	Creatine (300 mg/kg) orally for 4 weeks, starting 1 week prior to TBI induction.	Increased latency to first myoclonic and tonic–clonic seizures. Reduced time spent in tonic–clonic seizure. Decreased seizure intensity.
Okwuofu et al. (2021)	PTZ-kindled mice.	Creatine (75, 150, and 300 mg/kg/day, orally) for 15 days.	Reduced seizure severity scores, depression, and anxiety-like behaviors. Reduced oxidative stress markers.

IV, intravenous; PTZ, pentylenetetrazol; IP, intraperitoneal; TBI, traumatic brain injury.

Creatine supplementation was also shown to be protective following TBI in rodent model and humans. TBI is a common problem affecting around 10 million people worldwide each year (Hyder et al., 2007). This pathology causes long-term medical complications including recurrent spontaneous epileptic seizure, referred to as post-traumatic epilepsy (PTE) (Atkins et al., 2010). It has been suggested that the increased excitability leading to PTE following trauma is caused by progressive impairment in GABAergic function in the hippocampus (Pavlov et al., 2011). Studies have shown that treating TBI patients with creatine (400 mg/kg, orally) 4 hours after hospital admission and then daily for 6 months, presented an improved cognition, less reporting of headache, and an overall shorter period spent in intensive care units (Sakellaris et al., 2008). Experimental models of TBI in rodents have demonstrated that these neuroprotective effects of creatine are associated with its ability to reduce the spread of cortical damage (Scheff and Dhillon, 2004). Gerbatin et al. investigated the impact of delayed and chronic creatine supplementation (300 mg/kg, orally for 4 weeks, initiated 1 week following trauma) on susceptibility to epileptic seizures induced by PTZ after TBI in rats. This study revealed that creatine supplementation notably increased the latency to first myoclonic and tonic–clonic seizures, decreased the time spent in tonic–clonic seizure, reduced seizure intensity, and decreased epileptiform discharges and spindle oscillations induced by PTZ. This protective effect persists for1 week even when creatine supplementation was discontinued (Gerbatin et al., 2019). Furthermore, creatine supplementation also protected against cell loss including GABAergic neurons associated with TBI (Table 1; Gerbatin et al., 2019). However, in another study performed on the same rat model of TBI, supplementing the rats with 300 mg/kg creatine daily for 7 days following trauma reduced oxidative stress markers but did not protect against seizure susceptibility following severe TBI (Saraiva et al., 2012).

Recently, the ameliorative effects of creatine supplementation on seizure severity and behavioral changes were evaluated in PTZ-kindled mice as a model of chronic epilepsy (Okwuofu et al., 2021). Kindling is a process of inducing progressive intensity of convulsive activity as a result of repetitive administration of chemical or electrical sub-convulsive stimuli (Pavlova et al., 2004). In this study, creatine supplementation (75, 150, or 300 mg/kg, orally for 15 days) significantly reduced seizure severity scores, anxiety, and depression-like behaviors in mice from the 5^th^ day of treatment. In addition, creatine significantly increased catalase and glutathione activities and decreased lipid peroxidation when compared to vehicle treated PTZ-kindled mice suggesting antioxidant activity of this compound (Table 1; Okwuofu et al., 2021).

Since creatine supplementation was shown to have anticonvulsant properties in rodent models of seizure, a recent study investigated whether creatine supplementation would provide added beneficial effects on seizure control when combined with ketogenic diet in children (Kalamitsou et al., 2019). It is well known that ketogenic diet has antiepileptic effects that could be attributed to increase in ATP and PCr levels and consequent improvement of promotion of energy balance within the CNS (Ulamek-Koziol et al., 2019). While ketogenic diet is effective in some cases, not all patients benefit from it; in most cases this differential response to the diet is not well understood. In the study by Kalamitsou et al., their rational was that increasing PCr: creatine energy store ratio by creatine supplementation could enhance the efficacy of the ketogenic diet and increase its clinical benefits in children (10 months – 8 years old) with refractory epilepsy (Kalamitsou et al., 2019). All 22 children recruited for this study were on ketogenic diet and oral creatine was administered at a dose of 0.4 g/kg, daily, divided into two doses. Creatine supplementation produced a complete cessation of seizure in 2 patients and a 70 to 90% reduction in seizure frequency was reported in additional five patients. Although the data involve a small cohort, creatine effect was either positive or neutral (Kalamitsou et al., 2019). However, future double-blind clinical trials with large sample size are needed to further explore the beneficial effects of creatine supplements on seizure.

### 2.5. What is the best dosing strategy for muscle and brain creatine loading?

The performance benefits that result from creatine supplementation include higher cellular energy for short, high-intensity exercise, improved energy transfer in muscle cells, greater buffering capacity resulting in less fatigue and shorter recovery time (Nelson et al., 2001; Cooke et al., 2009). Supplementation with CM for improved athletic performance typically involves a daily loading dose of 20 g (4 × 5 g) over a five-day period, followed by a maintenance dose of 3–5 g daily. Studies have demonstrated that this dosing strategy is correlated with an average increase in total skeletal muscle creatine levels by approximately 20% (Harris et al., 1992). The observed increase in creatine in skeletal muscle was correlated with improved anaerobic exercise performance as measured by increased work output, strength, exercise capacity, and muscle mass (Wax et al., 2021).

While consistent information is available on supplementation protocols aimed at increasing muscle creatine content, much less is known regarding the optimal supplementation strategy to increase brain creatine levels, this is largely due to limited dataset that directly measures the concentration of creatine in the brain following supplementation (Roschel et al., 2021). Moreover, the large heterogenicity in respect to brain creatine assessment techniques, differences in creatine content in different regions of the brain, different creatine dosages and duration of supplementation, and variations in population characteristics, all hampers direct comparison between the few studies available on the topic (Rawson and Venezia, 2011). The available literature suggests possible increases in creatine in the brain following supplementation, though smaller than that seen in muscle (about 3%–10% vs. 20% increase in muscles from baseline) (Pan and Takahashi, 2007; Kondo et al., 2011; Turner et al., 2015). The variation in muscle and brain uptake of creatine could be explained by several reasons. Skeletal muscle does not have the ability to synthesize creatine, however, more than 95% of creatine is stored in muscles. Creatine obtained from endogenous sources or through the diet enters skeletal muscle via CRT1 (Forbes et al., 2021). On the other hand, the brain has the ability to synthesize creatine and appears to be more resistant to the uptake of circulating creatine due to lack of expression of CRT1 in the astrocytes involved in the BBB (Beard and Braissant, 2010). It appears that the brain could be solely relying on endogenous creatine synthesis until there is some sort of challenge to brain creatine content, in which case exogenous creatine supplementation could be of great value. Therefore, creatine ingestion may need to be higher or for longer periods of time to produce beneficial effects in the brain compared to skeletal muscle (Dechent et al., 1999). Current literature suggests a need for high dose and long duration supplementation protocol (i.e., 20 g/day for 4 weeks) to increase brain creatine levels (Dechent et al., 1999).

### 2.6. Limitations for creatine monohydrate supplementations

Despite promising potential of creatine, there are important limitations to be considered, including oral bioavailability and BBB permeability. The current commercially available form of creatine is CM, and it is the form used in animal studies and human clinical trials in the literature. As mentioned before, exogenously administered creatine must first be absorbed into the bloodstream across the intestinal wall and then taken up by CRT1 to be stored or utilized by the various tissues (Jomura et al., 2022). To date, CM supplementation has shown strong beneficial effects in many *in-vitro* and animal models of neurodegenerative disorders. In contrast, most of the clinical trials in humans have failed to show beneficial effects of creatine (Forbes et al., 2022), however, clinical trials in patients with seizure disorders are lacking. The lack of beneficial effects might be due to the doses used in most of the human studies, which were substantially lower than the allometric dose equivalent given in the preclinical studies. In most of the successful animal studies, CM was given at doses ranging from approximately 0.2 to 0.6 g per day. Based on allometric dosing, this would require a 20–40 g daily dose in human studies, which is substantially larger than the doses used in most of the clinical trials. In addition, it is known that the aqueous solubility of CM is around 16.6 mg/mL (Gufford et al., 2010). Since it is usually given at high doses (5–20 g/day for athletes, and > 25 g/day for therapeutic applications), it is likely administered as an oral suspension. As only the solubilized creatine is available for absorption in the intestines, such a dosing practice would suggest that a substantial portion of the creatine consumed is unabsorbed. While definitive oral bioavailability data for CM in humans is lacking, a study by Alraddadi et al., examining CM oral bioavailability in rats suggests CM oral bioavailability is at most 48% (Alraddadi et al., 2018). The relatively large doses of creatine required to produce the desired therapeutic effects suggests inefficiencies in either bioavailability and/or tissue distribution of current creatine products. This highlights the importance of finding alternative creatine forms with improved and more efficient dosage formulations.

## 3. Conclusions and future directions

Over the last years, numerous AEDs have been developed and approved for the management of epilepsy, however, the symptoms in many patients remain uncontrolled even when a combination of drugs are used. The evidence discussed in this review suggest the involvement of impaired brain bioenergetics in the development of epilepsy. Candidate compounds with the potential to target brain energetics have promising future in the management of epilepsy and other related neurological disorders. Creatine is a naturally-occurring organic acid that serves as an energy buffer and energy shuttle in tissues, such as brain and skeletal muscle, that exhibit dynamic energy requirements. Currently, limited evidence mainly from preclinical studies suggests anticonvulsant properties of creatine; however, the exact mechanism remain to be elucidated. Future work should involve larger clinical trials of creatine used as an add-on therapy, followed by large clinical trials of creatine as monotherapy. However, before moving forward with human clinical trials, it is important to first determine the optimal creatine protocol for increasing brain creatine to the levels in which beneficial effects of creatine could be reached. So far, dose–response studies are lacking, and the protocols used in the literature for supplementation and measurements are heterogenous. Secondly, the identification of new and improved forms of creatine with improved aqueous solubility, oral bioavailability, and tissue distribution is needed. Currently, there is increased intertest in different salt forms of creatine with improved solubility parameters (Gufford et al., 2010). Of the newer salt forms of creatine, there are three: creatine hydrochloride (CHCl) creatine pyruvate (CrPyr), and creatine citrate (CrC), for which human oral bioavailability have been reported to be higher than that of CM (Jager et al., 2007). This suggests the potential for development of creatine supplements with improved oral absorption, which in turn could provide significant advancements in performance benefits, and allow for reduced dosages and more flexible dosing formulations.

## Author contributions

All authors listed have made a substantial, direct, and intellectual contribution to the work and approved it for publication.

## Conflict of interest

The authors declare that the research was conducted in the absence of any commercial or financial relationships that could be construed as a potential conflict of interest.

## Publisher’s note

All claims expressed in this article are solely those of the authors and do not necessarily represent those of their affiliated organizations, or those of the publisher, the editors and the reviewers. Any product that may be evaluated in this article, or claim that may be made by its manufacturer, is not guaranteed or endorsed by the publisher.
